# G-Protein Coupled Receptor Kinase 2 Minimally Regulates Melanopsin Activity in Intrinsically Photosensitive Retinal Ganglion Cells

**DOI:** 10.1371/journal.pone.0128690

**Published:** 2015-06-12

**Authors:** Timothy J. Sexton, Russell N. Van Gelder

**Affiliations:** 1 Department of Ophthalmology, University of Washington School of Medicine, Seattle, Washington, United States of America; 2 Program in Neurobiology and Behavior, University of Washington School of Medicine, Seattle, Washington, United States of America; 3 Department of Pathology, University of Washington School of Medicine, Seattle, Washington, United States of America; 4 Department of Biological Structure, University of Washington School of Medicine, Seattle, Washington, United States of America; Morehouse School of Medicine, UNITED STATES

## Abstract

Phosphorylation is a primary modulator of mammalian G-protein coupled receptor (GPCR) activity. The GPCR melanopsin is the photopigment of intrinsically photosensitive retinal ganglion cells (ipRGCs) in the mammalian retina. Recent evidence from *in vitro* experiments suggests that the G-protein coupled receptor kinase 2 (GRK2) phosphorylates melanopsin and reduces its activity following light exposure. Using an ipRGC-specific GRK2 loss-of-function mouse, we show that GRK2 loss alters melanopsin response dynamics and termination time in postnatal day 8 (P8) ipRGCs but not in older animals. However, the alterations are small in comparison to the changes reported for other opsins with loss of their cognate GRK. These results suggest GRK2 contributes to melanopsin deactivation, but that other mechanisms account for most of modulation of melanopsin activity in ipRGCs.

## Introduction

Intrinsically photosensitive retinal ganglion cells (ipRGCs) are photoreceptors in the mammalian retina which mediate photic entrainment of circadian rhythms and control the pupillary light response [1]. These cells use melanopsin (Opn4) as their intrinsic photopigment [2, 3]. Like rods and cones, ipRGCs exhibit adaptation changes, including desensitization under continuous background light, ‘resensitization’ to flashes of light during the decaying phase of a continuous light response, acceleration of light responses under continuous illumination, and recovery from desensitization upon extended dark incubation [4]. Recently, Do and Yao [5] have shown that ipRGC responses follow a Weber-Fechner-like relation [6] upon exposure to increasing background light levels similar to rods and cones.

The mechanisms underlying ipRGC adaptation are not well understood, but may be similar to those in rods and cones. Following light activation rhodopsin and cone-opsins are phosphorylated on C-terminus residues by a G-protein-coupled receptor kinase (GRK). Phosphorylation is followed by binding of an arrestin molecule to inactivate the opsin [6, 7]. Currently 7 subtypes of GRKs are known: GRK1-GRK7. Rods express GRK1 (rhodopsin kinase) and cones express GRK7 (cone-opsin kinase) [8, 9].

Several studies have demonstrated melanopsin phosphorylation following light exposure in both heterologous expression systems and in ipRGCs [10–12]. Phosphorylation of C-terminal serine/threonine residues alters melanopsin response kinetics when expressed in HEK 293 cell line [10]. GRK2 has been implicated in this phosphorylation control of melanopsin activity [11]. GRK2 and GRK3 can bind melanopsin *in vitro* and regulate melanopsin-mediated intracellular calcium changes in HEK293 cells expressing melanopsin [11]. GRK2 and GRK3 colocalize with melanopsin expression in ipRGCs [11]. Here we expand on this work by testing ipRGC responses in a mouse line with ipRGC-specific GRK2 loss-of-function. Changes in ipRGC light responses were minimal.

## Methods

### Animals

All experiments were performed in accordance with Association for Research in Vision and Ophthalmology guidelines for animal studies, under an approved animal study protocol from the Institutional Animal Care and Use Committee of University of Washington (Protocol #4184–01). Animals were maintained in a 12:12-hr light-dark cycle with *ad libitum* food. Animals with a floxed GRK2 gene (hereafter GRK2^flox^) in a B6/129 background were purchased from Jackson Laboratory (Adrbk1^tm1Gwd^/J, Jackson Labs, Bar Harbor, Maine). GRK2^flox^ mice were bred with a line of C57BL/6, 129SvJ mixed background mice containing a Cre-recombinase gene knocked into the melanopsin locus (Gift of Samer Hattar, [13]). Experimental animal genotypes were Cre^+/-^ / GRK2^flox/flox^ for knockouts and Cre^+/-^ / GRK2 ^+/+^ for controls. Animals were used at P8-P10 (P8) and P30-P35 (P30) and were littermates or progeny of the same set of breeding pairs for repeated experiments.

### Preparation of Retina

Mice were dark-adapted for 1-hr prior to experiments, and all subsequent procedures were performed under dim red light illumination. All experiments were performed between noon and 6 pm (Circadian time [CT] 5–11) except for 1 minute light recovery experiments, which were performed between 9 pm and midnight (CT 14–17). Mice were sacrificed with CO_2_ euthanasia and cervical dislocation. Retinas were isolated in a bicarbonate-buffered physiologic solution (125 mM NaCl, 2.5 mM KCl, 1 mM MgCl_2_, 1.25 mM NaH_2_PO_4_, 20 mM glucose, 26 mM NaHCO_3_, 2 mM CaCl_2_, 500 μM glutamine) oxygenated with 95% O_2_/5% CO_2_ to obtain a pH of 7.4. Isolated mouse retinas were cut in half, positioned with the vitreal face in contact with a multielectrode array (MEA) and superperfused at 2–3 ml/min with the bicarbonate-buffered physiologic solution. Perfusate and tissue chamber temperature was maintained at 33.0°C. In P8 retinas, the outer retina does not form functioning synaptic input to the inner retina; however, spontaneous retinal waves are prevalent [14]. These were suppressed with a cholinergic inhibitor (5 nM epibatidine) and glutamatergic inhibitors [50 μM d(2)-2-amino-5-phosphonopentanoic acid (d-AP5); 20 μM d(-)-2-amino-4-phosphonobutyric acid (d-AP4), 10 μM CNQX] (Tocris Biosciences, Ellisville, MO). In recordings from P30 retinas, outer retinal input was suppressed with glutamatergic inhibitors (200 μM d-AP5, 100 μM d-AP4 and 80 μM CNQX).

### Multi-electrode recordings and light stimulation

MEA recordings were performed using planar arrays of 60 electrodes (30 μm diameter, 200 μm inter-electrode spacing; Multi Channel Systems, Reutlingen, Germany). Raw electrical signals were amplified, filtered, and digitized through an A/D card (National Instruments, Austin, TX), written to disk and analyzed off-line, as described previously [15].

For continuous light exposures, a Xenon light source (Sutter Instruments, Novato, CA) fed through a liquid light guide and diffusing filter (Thorlabs Inc., Newton, NJ) was used. Intensities and wavelengths of light were adjusted by neutral density and narrow band-pass 480 nm interference filters respectively (Thorlabs, Inc., Newton, NJ). Light intensity was measured with a radiometer (Advanced Photonics International, Fairfield, CT). Briefly, retinas were successively exposed to 10-sec, 30-sec and 60-sec of continuous 480 nm at 3.98 x 10^13^ photon cm^-2^ s^-1^ (IR 13.6, where IR corresponds to log_10_ of the irradiance in units photon cm^-2^ s^-1^). This was followed by 1, 3 and 5 brief flashes of bright white light (4.6 J m^-2^) from a commercial camera flash (SunPak). Multiple flashes were 5-sec apart (fastest recharge time of flash). The 3 flash experiments are not reported here for brevity. Data for individual response parameters are represented in boxplots.

### ipRGC Subtype Classification

Categorizing ipRGC subtypes was done as previously described [16] with modifications to reduce light exposure prior to recovery experiments. Subtyping measurements were made at the beginning of experiments. P8 cells were first divided into cells with on-latencies, time from light on to time of peak firing, of less than 12-sec and greater than 12-sec in response to a 1-min, 480 nm light at an irradiance of 1.0 x 10^12^ photon cm^-2^ s^-1^ (log_10_ (1.0 x 10^12^ photon cm^-2^ s^-1^) = IR 12.0). Cells with on-latencies less than 12-sec were designated Type III cells. Cells with on-latencies greater than 12-sec were further divided. Those with an IR 12.0 light response greater than 10% of their IR 13.6 (3.98 x 10^13^ photon cm^-2^ s^-1^) light response were designated Type I cells. Those cells with an IR 12.0 light response of less than or equal to 10% of their IR 13.6 response were categorized as Type II cells (Table 1). In P30 animals, cells were not subtypes because of the small number of cells obtained.

**Table 1:** ipRGC functional subtype categorization rules.

	Type I	Type II	Type III
**P8**	On-latency > 12-sec at IR 12.0	On-latency > 12-sec at IR 12.0	On-latency < 12-sec at IR 12.0
	Light response at IR 13.6 and 12.0	Light response at IR 13.6 and response at 12.0 of < 10% 13.6 response	

### Pharmacology

Retinas from wildtype C57BL/6 mice were treated with the GRK2/5/6 inhibitor bisindolylmaleaimide XI hydrochloride (Adipogen, San Diego, CA). Because of low solubility, the inhibitor required a 1% DMSO carrier. Under synaptic blockade, retinal light responses were assessed with flashes of white light. P8 retinas were tested with 5 flashes and P30 retinas were tested with 1 flash. Retinas were then treated with 1% DMSO in bubbling AMES for 10-min, washed in AMES for 5-min, re-exposed to synaptic blockade for 5-min, and tested for ipRGC light responses. Finally, retinas were treated with the GRK inhibitor (100–200 μM in bubbling AMES with 1% DMSO) for 10-min, washed in AMES for 5-min, re-exposed to synaptic blockade for 5-min and tested for ipRGC light responses.

### Recovery

In recovery experiments, ipRGCs were exposed to 1-min of 480 nm light at 3.98 x 10^13^ photon cm^-2^ s^-1^ (IR 13.6), allowed to recover for intervals of 1, 3, 5, or 10 minutes in the dark and retested with a 1-min exposure of the same light. Percent recovery is calculated as the number of spikes during light in the second exposure of an interval divided by the number of spikes in the first exposure of the interval.

## Results

**Fig 1:**
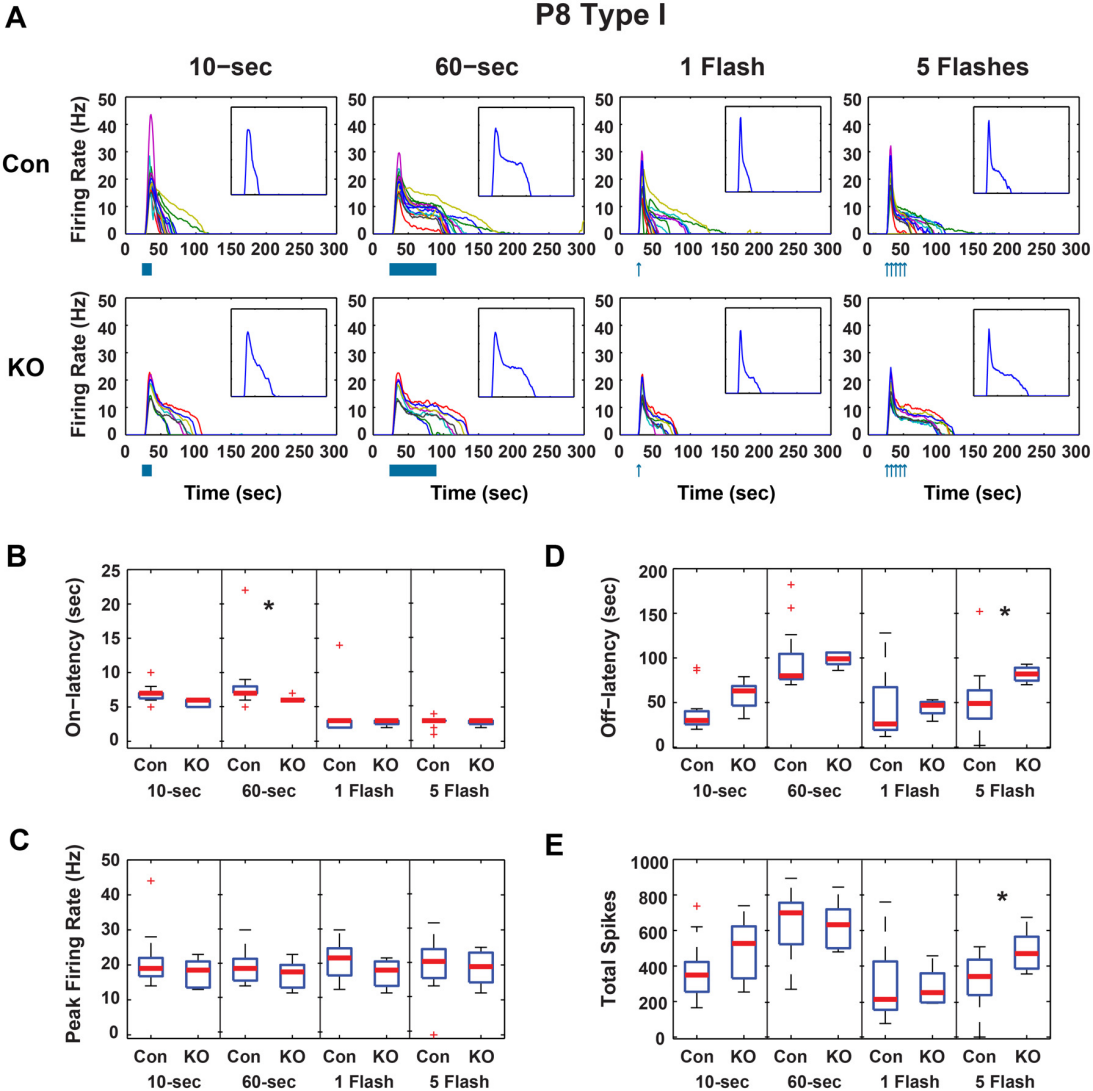
GRK2^flox/flox^;Opn4^Cre^ P8 Type I ipRGC light responses. A) Firing rates for individual P8 Type I control (CON, n = 15) and GRK2^flox/flox^;Opn4^Cre^ (KO, n = 8) ipRGCs in response to 10-sec and 60-sec continuous light and 1 and 5 flashes of light. Inset is the median firing rate for all cells in the larger plot. Boxplots of response parameters for P8 Type 1 cells including B) On-latency, C) Peak Firing, D) Off-latency, and E) Total spikes. * Wilcoxon rank sum, *p* < 0.05, Bonferroni corrected for 4 comparisons).

**Fig 2:**
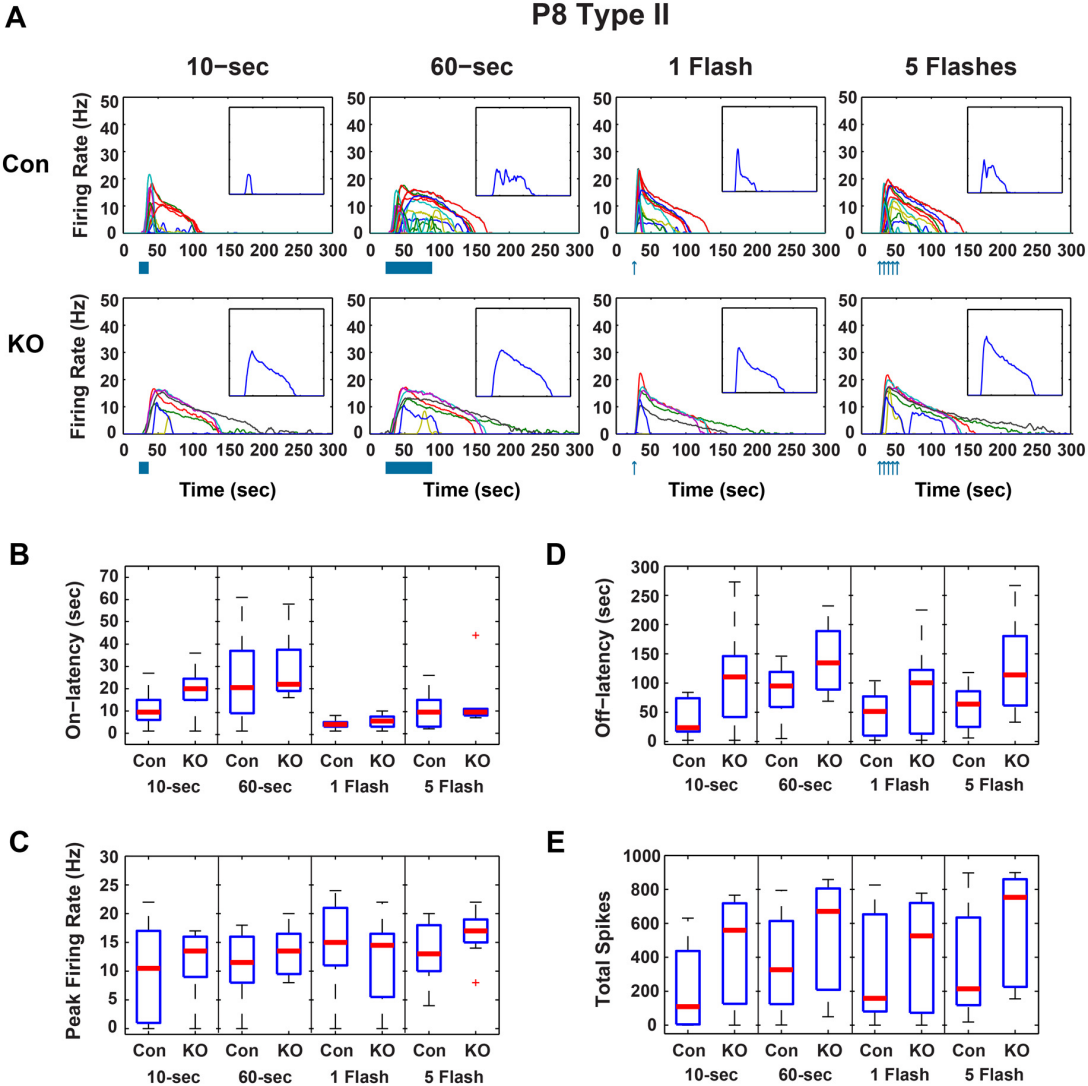
GRK2^flox/flox^;Opn4^Cre^ P8 Type II ipRGC light responses. A) Firing rates for individual P8 Type II control (CON, n = 18) and GRK2^flox/flox^;Opn4^Cre^ (KO, n = 8) ipRGCs in response to 10-sec and 60-sec continuous light and 1 and 5 flashes of light. Inset is the median firing rate for all cells in the larger plot. Boxplots of response parameters for P8 Type II cells including B) On-latency, C) Peak Firing, D) Off-latency, and E) Total Spikes. * Wilcoxon rank sum, *p* < 0.05, Bonferroni corrected for 4 comparisons).

**Fig 3:**
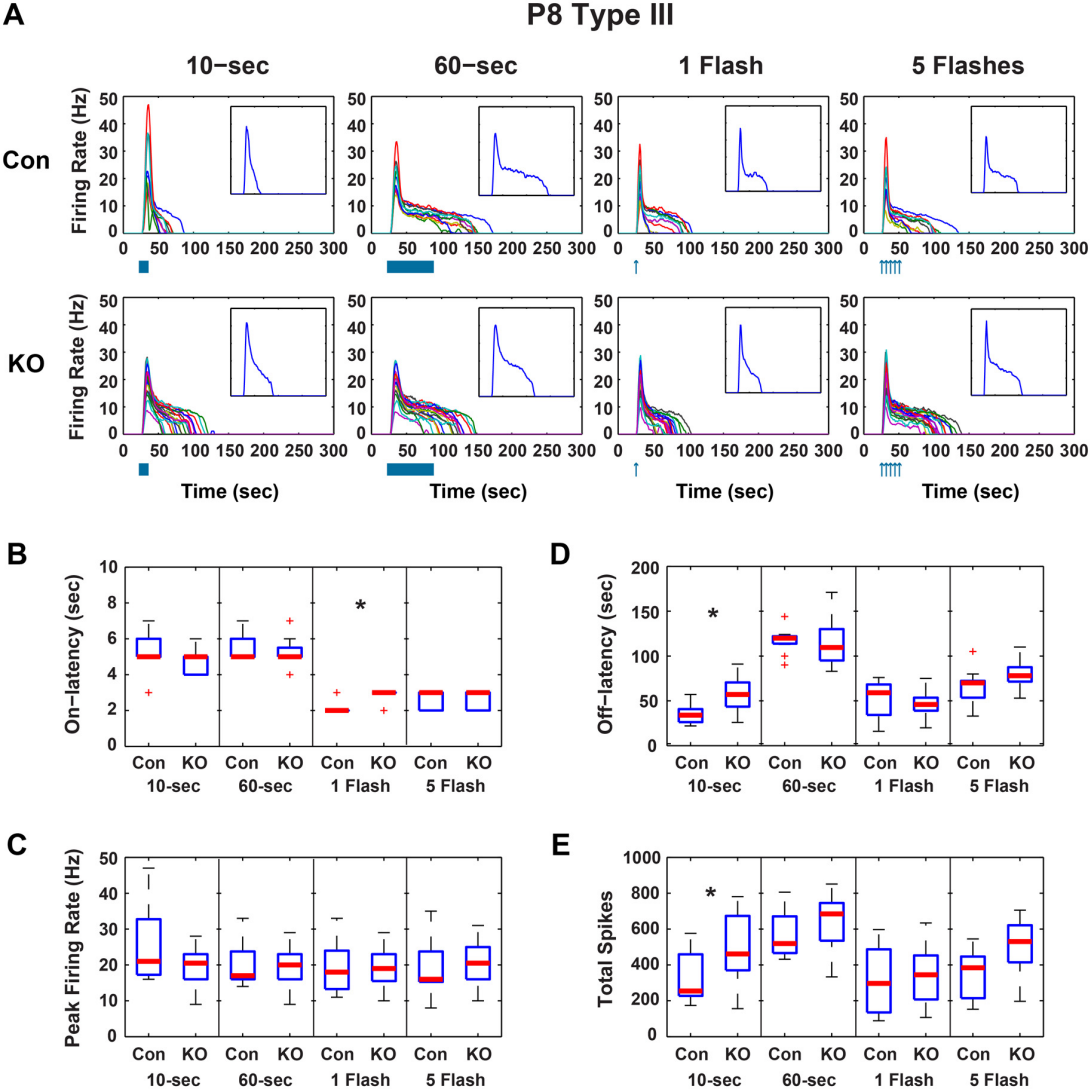
GRK2^flox/flox^;Opn4^Cre^ P8 Type III ipRGC light responses. A) Firing rates for individual P8 Type III control (CON, n = 11) and GRK2^flox/flox^;Opn4^Cre^ (KO, n = 18) ipRGCs in response to 10-sec and 60-sec continuous light and 1 and 5 flashes of light. Inset is the median firing rate for all cells in the larger plot. Boxplots of response parameters for P8 Type III cells including B) On-latency, C) Peak Firing, D) Off-latency, and E) Total Spikes. * Wilcoxon rank sum, *p* < 0.05, Bonferroni corrected for 4 comparisons).

In GRK^flox/flox^;Opn4^Cre^ P8 cells, peak firing was unchanged from controls (Fig 1), indicating the ability of cells to fire was intact in animals lacking GRK2 activity in ipRGCs. Significant changes in P8 Type I cells of these mice included increases in the off-latency (time from lights off to end of firing in the case of the a continuous exposures or time from light flash until the end of firing) from a median of 49 to 82-sec and a corresponding increase in the total number of spikes from a median of 341 to 470 spikes when exposed to a 5-flash stimulus (*p* < 0.05, Wilcoxon rank sum, Bonferroni corrected) (Fig 1). No significant changes were seen in P8 Type II cells (Fig 2). In Type III cells, there were significant increases over control in median off-latency (34 to 57-sec) and total spikes (255 to 462 spikes) in response to a 10-sec light pulse. The on-latency was increased from 2-sec to 3-sec after a single flash (*p* < 0.05, Wilcoxon rank sum, Bonferroni corrected)(Fig 3). Decay times were calculated for a single flash exposure in all cells (time from peak firing to 33% peak firing). Though median P8 decay times were longer in GRK^flox/flox^;Opn4^Cre^ than in control animals, these changes were not significant.

**Fig 4:**
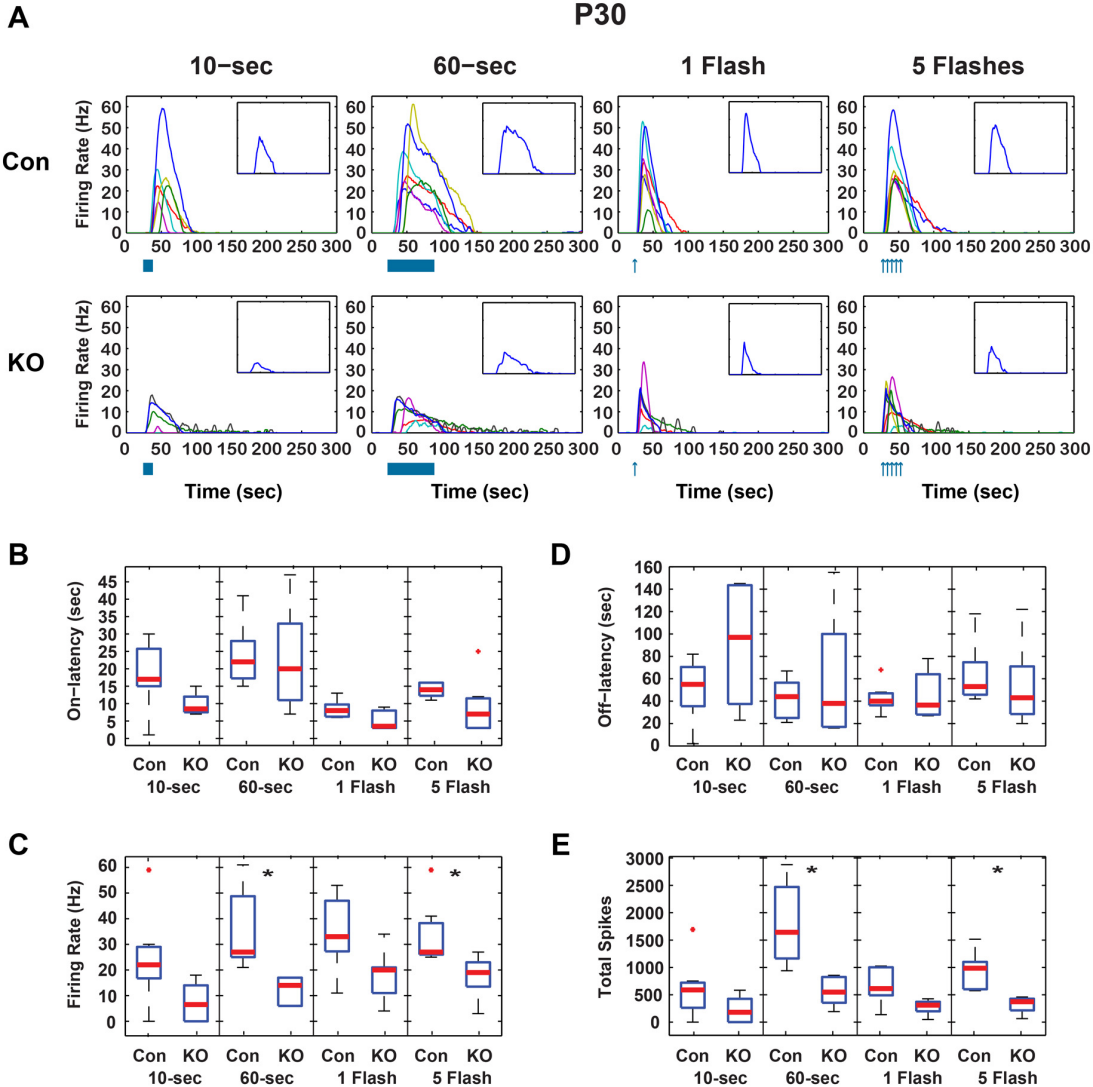
GRK2^flox/flox^;Opn4^Cre^ adult ipRGC light responses. A) Firing rates for individual adult control (CON, n = 7) and GRK2^flox/flox^;Opn4^Cre^ (KO, n = 6) ipRGCs in response to 10-sec and 60-sec continuous light and 1 and 5 flashes of light. Inset is the median firing rate for all cells in the larger plot. Boxplots of response parameters for P30 cells including B) On-latency, C) Peak Firing, D) Off-latency, and E) Total Spikes. * Wilcoxon rank sum, *p* < 0.05, Bonferroni corrected for 4 comparisons).

**Fig 5:**
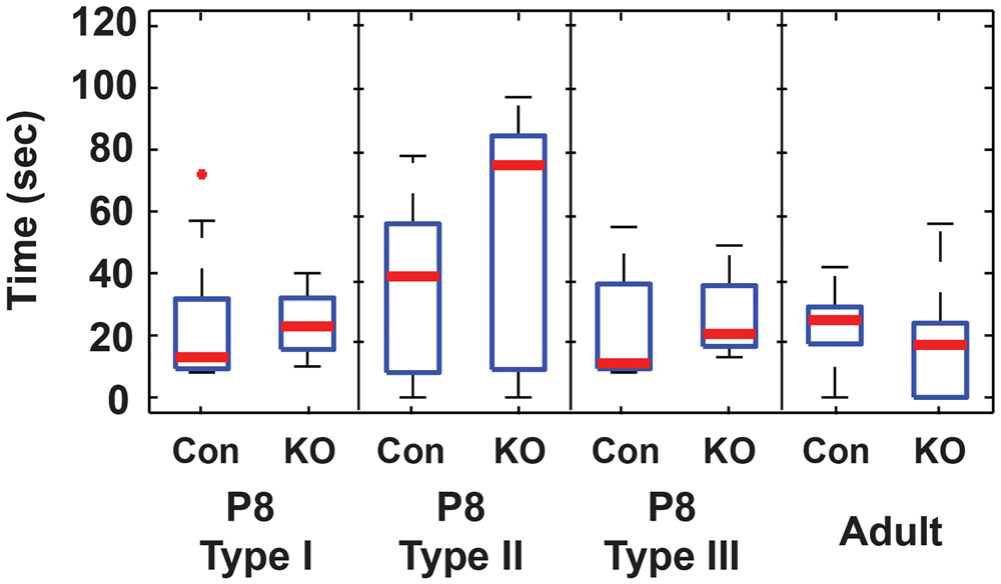
Decay times following a single flash are unchanged in GRK2^flox/flox^; Opn4^Cre^ ipRGCs. Con: control, KO: Knockout. Central lines represent medians, boxes indicate the interquartile range (25–75%), and whiskers extend to the range of data not considered outliers. Outliers, represented with a +, are data points that correspond to a distance greater than 2.7 standard deviations from the mean assuming the data is normally distributed.

In P30 ipRGCs, median peak firing was reduced in GRK^flox/flox^;Opn4^Cre^ vs. control under all conditions (Fig 4). These reductions were only significant under the 60-sec exposure (27 to 14 Hz) and 5-flash exposure (27 to 19 Hz). Similarly, median total spikes decreased significantly under the 60-sec exposure (1643 to 548 spikes) and 5-flash exposure (986 to 373 spikes)(*p* < 0.05, Wilcoxon rank sum, Bonferroni corrected). On- and off-latencies were not significantly different from controls. Median decay times in P30 cells were also unchanged (Fig 5).

**Fig 6:**
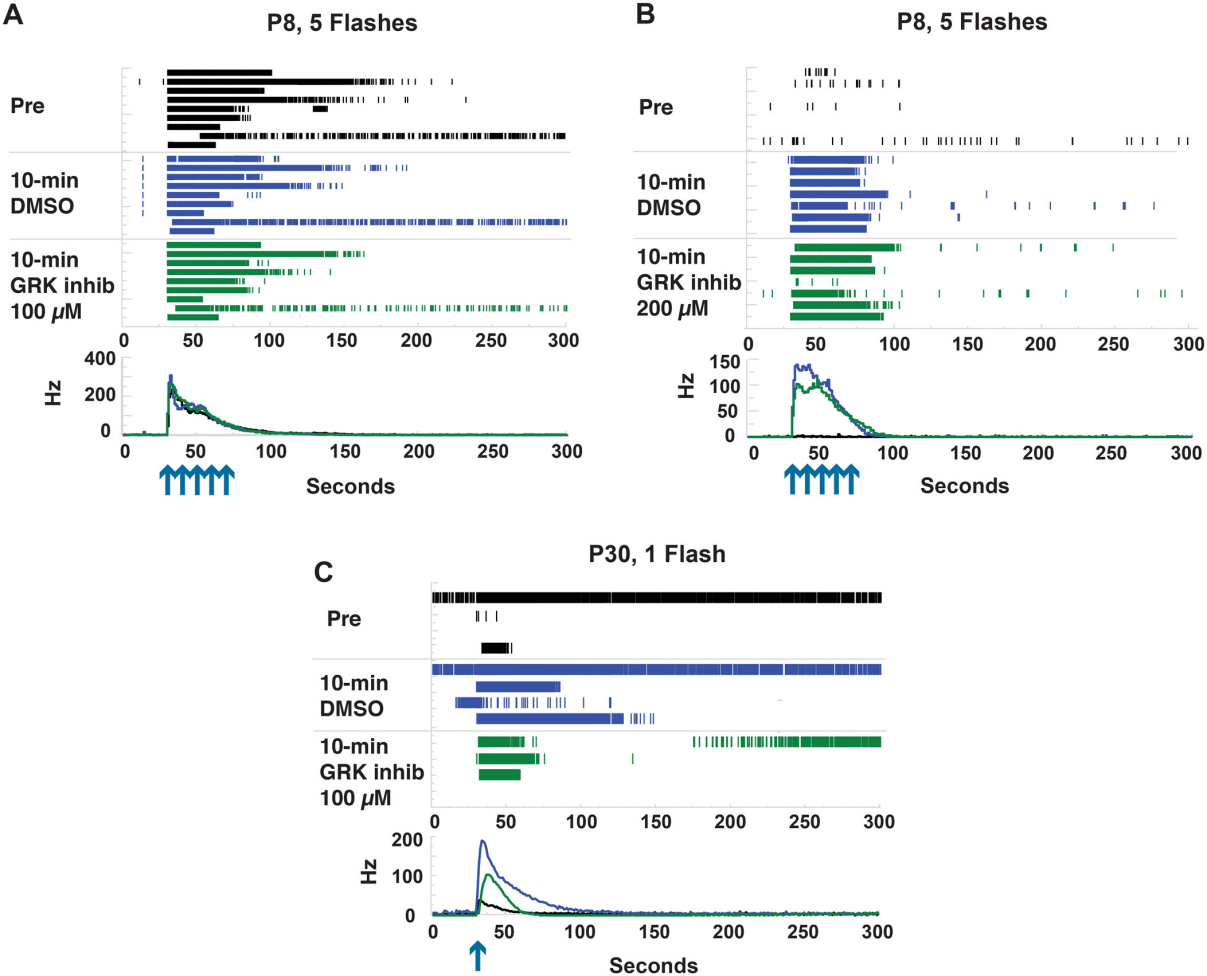
Treatment of ipRGCs with the GRK2/5/6 inhibitor bisindolylmaleimide XI. Light response measurements were made pre-treatment, post-DMSO carrier control treatement, and post-inhibitor treatment. Raster plots of A) 9 P8 ipRGCs treated with 100 μM inhibitor, B) 7 P8 ipRGCs treated with 200 μM inhibitor, C) 4 P30 ipRGCs treated with 100 μM inhibitor. Arrows indicate flashes of white light.

Treatment with the GRK2/5/6 inhibitor bisindolylmaleimide XI did not phenocopy GRK^flox/flox^;Opn4^Cre^ ipRGC response dynamics in P8 animals. The EC_50_ of bisindolylmaleimide XI for GRK2 inhibition in cell culture is 30 μM [17]. As changes in ipRGC activity were not observed with 30 μM treatment, 100 and 200 μM concentrations were used in subsequent experiments. No substantial change in responses were observed compared with those of the 1% DMSO control (Fig 6A and 6B). A 1% DMSO solution was the minimum DMSO concentration achievable because of low inhibitor solubility. Interestingly 1% DMSO did increase cell photosensitivity over pre-treatment levels in some cases (Fig 6B and 6C). This was often accompanied by higher baseline firing. The higher baseline which would reflect a more depolarized state of the cells and could explain the increased light response.

Treatment of retinas of P30 animals with 100 μM bisindolylmaleimide XI decreased ipRGC response as in the P30 GRK^flox/flox^;Opn4^Cre^. However, these decreases were greater under drug treatment (Fig 6). Further confounding interpretation, bisindolylmaleimide XI is known to be a GRK5/6 and protein kinase C inhibitor at concentrations 10-fold lower than for GRK2 inhibition [17].

**Fig 7:**
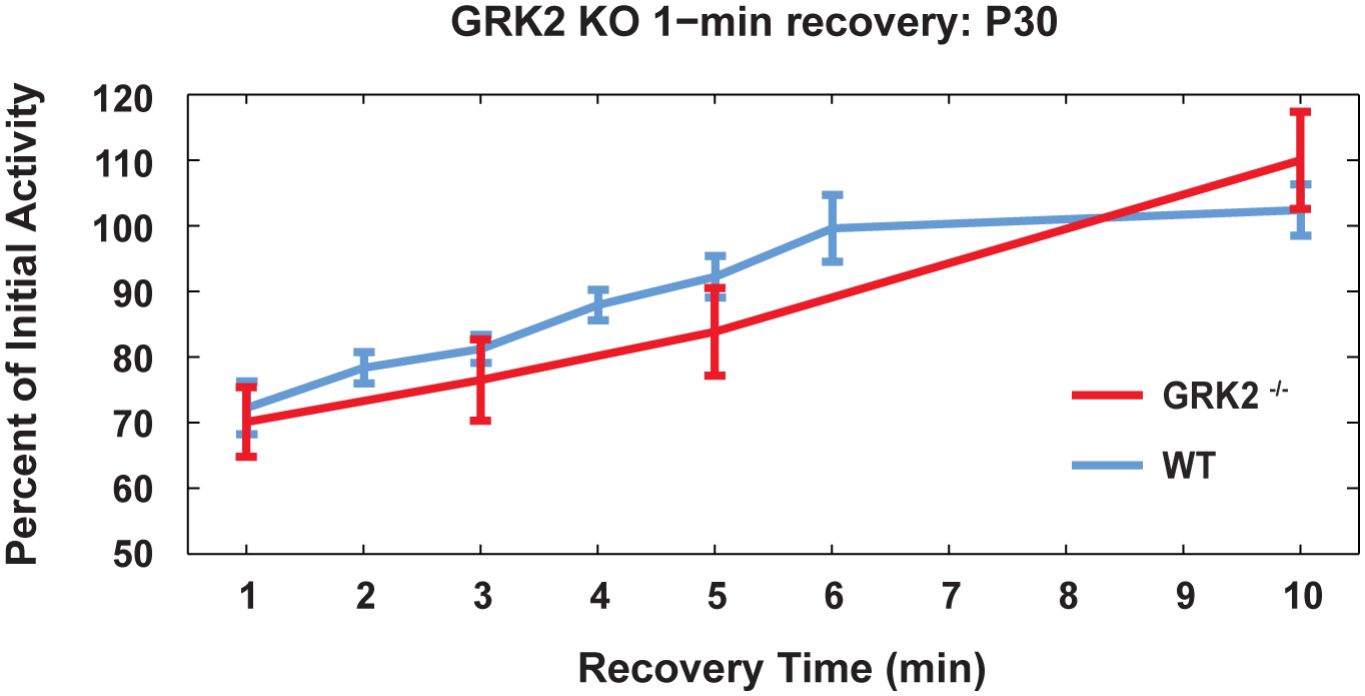
Time course of P30 wildtype (CON, n = 6 to 16) and P30 GRK2^flox/flox^;Opn4^Cre^ (KO, n = 7) ipRGC recovery following a 1-min light exposure. Retinas were tested with one minute light exposure at baseline, and again with identical light stimulus at time indicated. Total firing in one minute was calculated as percentage of baseline and plotted.

For other GPCRs, GRK2 influences recovery from desensitization and receptor internalization [18]. Therefore P30 GRK^flox/flox^;Opn4^Cre^ ipRGCs were tested for changes in recovery from a 1-min 480 nm light at 3.98 x 10^13^ photons s^-1^ cm^-2^ (IR 13.6). Recovery in GRK^flox/flox^;Opn4^Cre^ ipRGCs overlapped with wildtype aged matched ipRGCs (Fig 7).

## Discussion

Removing GRK2 from ipRGCs had a limited effect on ipRGC responses. Only off-latencies, and total spikes increased significantly in P8 ipRGCs. This would be expected by removing phosphorylation inactivation mechanisms. However, the changes are small in comparison to the changes in rod responses in mice lacking GRK1 [19]. That manipulation leads to a doubling of the rod single photon current response and an extension of response termination time by several hundred-fold. In P30 animals, peak firing rate and total spiking was reduced (the opposite effect to that expected from inhibiting melanopsin inactivation). Thus, GRK2 is likely not the sole mechanism terminating melanopsin activity.

The effect of bisindolylmaleimide XI on ipRGCs is difficult to interpret because it is also a potent protein kinase C inhibitor. At high concentrations it may also have non-specific toxic effects. Unfortunately, without a specific GRK2 inhibitor we cannot definitively say that the changes in GRK^flox/flox^;Opn4^Cre^ ipRGC kinetics are a direct consequence of reduced melanopsin phosphorylation, as opposed to a compensatory change resulting from loss of GRK2 that is not directly related to melanopsin.

In the work of Blasic et al., [20], both GRK2 and GRK3 bound to melanopsin in immunoprecipitation assays. However, when both were knocked down with siRNA GRK2 only was shown to alter melanopsin activity in cell culture. Using single cell RT-PCR, this group also found that GRK2 was present in all ipRGCs while GRK3 was found in only half of ipRGCs. Despite this, GRK3 regulation of melanopsin cannot be ruled out. That GRK3 is found in half of ipRGCs may reflect differential regulation in different ipRGC subtype. In GRK2^-/-^ ipRGCs, up-regulation of GRK3 could compensate for the absence GRK2. Such a dissociation between *in vitro* and *in vivo* results has also been seen in assessments of melanopsin G-protein utilization, where *in vitro* data strongly suggests a Gq/11-mediated transduction mechanism [21] while conditional knockouts of specific G-proteins suggests neither is necessary for normal melanopsin function [22].

Two hypotheses may explain the P8 GRK2 knockout data. First, other GRKs found in ipRGCs, such as GRK3, may phosphorylate the same sites on melanopsin as GRK2 but with lower efficiency. This would lead to a slower melanopsin inactivation, possibly reflected in the P8 results of this study. In the second hypothesis, GRK2 could be one of several GRKs coordinating melanopsin phosphorylation. Removing GRK2 may prevent phosphorylation of specific serine/threonine residues on the melanopsin C-terminus leading to changes in kinetics. Such differential phosphorylation by different GRK subtypes in other systems leads to differential receptor-arrestin localization within cells [8]. In cultured heart myocytes expressing peptide inhibitors of either GRK2 or GRK3, distinct subsets of receptors are modified depending upon the GRK type inhibited [23]. Alternatively, other non-GRK kinases may regulate melanopsin activity. For instance, PKA can phosphorylate melanopsin residues in cytoplasmic loops, which can reduce light activation of melanopsin [11]. Also, recent work by Fahrunkrug et al., (2014)[12], found little evidence for GRK2 or GRK3 mediated phosphorylation of specific C-terminal serine/threonine residues of melanopsin but did find evidence for PKC and SGK1 involvement *in vitro*. Additionally, it is conceivable that there are circadian rhythms to GRK function on melanopsin; we have not addressed this possibility in the current experiments.

Using an ipRGC-specific GRK2 knockout animal, we find evidence for a GRK2 component of melanopsin regulation in ipRGCs, particularly in neonatal mice. However, regulation of melanopsin activity does not appear to strictly follow the models suggested by mammalian rhodopsin and cone-opsins; it does not appear that GRK2 is the exclusive kinase attenuating melanopsin signaling. Other mechanisms remain to be elucidated.
